# What the visual system can learn from the non-dominant hand: The effect of graphomotor engagement on visual discrimination

**DOI:** 10.3758/s13421-024-01628-2

**Published:** 2024-11-05

**Authors:** Shlomit Ben-Ami, Batel Buaron, Ori Yaron, Kyle Keane, Virginia H. Sun, Flip Phillips, Jason Friedman, Pawan Sinha, Roy Mukamel

**Affiliations:** 1 Sagol School of Neuroscience, Tel-Aviv University, Tel Aviv, Israel; 2 School of Psychological Sciences, Tel-Aviv University, Tel Aviv, Israel; 3 Department of Brain and Cognitive Sciences, Massachusetts Institute of Technology, Cambridge, MA USA; 4 Massachusetts General Hospital, Boston, MA USA; 5 MAGIC Center, Rochester Institute of Technology, Rochester, NY USA; 6 Department of Physical Therapy, Faculty of Medical and Health Sciences, Stanley Steyer School of Health Professions, Tel Aviv University, Tel Aviv, Israel; 7 School of Computer Science, University of Bristol, Bristol, UK

**Keywords:** Laterality, Motor control, Object recognition, Reading, Visual perception

## Abstract

Previous studies have demonstrated that engaging in graphomotor activity for creating graphemes can enhance their subsequent visual discrimination. This suggests a positive influence of the motor system on visual learning. However, existing studies have emphasized the dominant hand, which is superiorly dexterous in fine-motor movements. This near-exclusive focus prompts the inquiry of whether the observed perceptual facilitation is a general characteristic of the motor system, or specific to pathways controlling the skilled over-trained dominant hand. Furthermore, the mechanistic underpinning of visual facilitation from graphomotor training (i.e., the individual contribution of motor activity, temporal evolution of the visual trace, variability of visual output) remain unclear. To address these questions, we assessed visual discrimination capabilities of healthy right-handed participants (N = 60) before and after graphomotor or visual training. Contrary to our initial expectation, graphomotor engagement with the non-dominant hand did not yield additional benefits to visual learning beyond those attainable through visual training alone. Moreover, graphomotor training with the non-dominant hand resulted in visual discrimination improvements comparable to those of dominant hand training, despite the inherent differences between hands in motor performance and in the amount of improvement in shape tracing throughout training. We conclude that the motor components of graphomotor activity may not be critical for visual learning of shapes through tracing activity. Instead, our results are in agreement with the symbolic theoretical account, suggesting that basic shape features required for discrimination can be acquired through visual inspection alone, providing a perspective on the improvements observed in prior studies.

**Supplementary Information:**

The online version contains supplementary material available at 10.3758/s13421-024-01628-2.

## Introduction

A growing body of evidence demonstrates a significant impact of motor engagement on visual processing. For instance, the subjective perception of a visual event, such as its perceived speed (Dewey & Carr, 2013) as well as the neurophysiological responses it evokes (Mifsud et al., 2018; Stenner et al., 2014; Yon & Press, 2017) differ when the sensory event is the product of voluntary movement relative to an identical event from an external source. Furthermore, objective performance on perceptual tasks, including accurately detecting movement direction or temporal delays, is enhanced for sensory events that are actively triggered by the perceiver (Desantis et al., 2014; van Kemenade et al., 2016).

The impact of movement on visual perception extends beyond such discrete visual events to encompass continuous visuo-motor engagement, such as graphomotor activity involving the active manual production of a visual pattern through tracing, copying, handwriting, or drawing. The exploration of the impact of graphomotor activity on visual perception has predominantly centered around the study of handwriting and its consequential effects on literacy. Handwriting, which involves the production of graphemes serving as characters in written scripts (e.g., letters in the Greek alphabet, Han characters in Chinese), has been extensively investigated regarding its influence on the recognition of these linguistic symbols in the context of literacy development. These studies have demonstrated that handwriting can facilitate visual learning. Specifically, performance on visual discrimination of patterns (Longcamp et al., 2006; Zemlock et al., 2018), as well as their matching, recognition, and identification (Tan et al., 2005; Wiley & Rapp, 2021; for reviews, see Fernandes & Araújo, 2021; James, 2017) is reported to improve after handwriting training, more than after training using only visual information. Fewer studies have explored the effects of drawing (Adi-Japha & Freeman, 2001), which involves graphomotor production of visual representations (e.g., objects, shapes, etc.) on visual learning. These studies have revealed enhanced visual recognition (Wammes et al., 2019) and discrimination (Fan et al., 2018) of objects after drawing them.

Since writing and drawing require fine dexterous movements, the effects reported above have been almost exclusively studied using the dominant (mostly right) hand of study participants. To the best of our knowledge, the effect of graphomotor training with the non-dominant hand on perceptual (visual) improvements has not been examined. Therefore, it is not clear whether the advantage of graphomotor activity in visual learning is a general property of the motor system or rather specific to circuits controlling the dominant hand.

Several factors suggest that it is not obvious to predict if facilitation of visual learning from graphomotor engagement would generalize to the non-dominant hand. Functionally, the difference between an individual’s dominant versus non-dominant hands is most pronounced during fine-motor movements such as those required for graphomotor activity, likely due to the different roles of the two hands (Bagesteiro & Sainburg, 2002; Callaert et al., 2011; Sainburg, 2014). While the dominant hand is over-trained and automated for such tasks, the non-dominant hand is scarcely used for these tasks and typically shows inferior dexterity, reflected as reduced accuracy and steadiness of line forming, and leading to increased variability of the visual output (Sandve et al., 2019). Neuroimaging evidence indicates that the neural response of visual areas to visuo-motor training may be distinct between dominant and non-dominant hand engagement. This is reflected, for example, by substantial differences between the hands in activation of visual regions during acquisition of visuo-motor adaptation (greater activation when the non-dominant hand is trained) and during bilateral transfer and after-effects (greater activation when the dominant hand is trained; Kirby et al., 2019). Even when perceptual consequences of the activity of the two hands are kept identical, the evoked response in the visual cortex is sensitive to which hand (left vs. right) was used to trigger the event. In other words, during active generation of a visual output, different patterns of neural activity are evoked in response to identical visual events triggered by the right versus left hand (Buaron et al., 2020). Taken together, the behavioral differences between the hands and their differential impact on neural activity in the visual cortex suggest that there may be hand-dependent differences in visual learning following motor engagement. Note that any perceptual differences between use of the dominant versus non-dominant hand may be related to the prominent differences in fine-motor skill between the hands (including spatial accuracy, movement smoothness, and movement duration), and thus any examination would need to account for those differences. It would also be of interest to isolate the contribution of the motor component from the visual components of the graphomotor output, including the dynamic continuous evolution of the visual trace and the variability of visual information due to variability in motor output.

Towards these goals, the aims of the current study were to:

1. Examine whether facilitation of visual learning through graphomotor training, previously documented for the dominant hand, also holds for the non-dominant hand.
2. Disentangle the relative contributions to visual learning of the motor component versus visual components associated with graphomotor engagement, including dynamic evolution and variability of the visual trace.
3. Examine whether visual learning through graphomotor training differs between the non-dominant and dominant hands.

To this end, we assessed short-term (after 2-day training) and retention (1 week) gains in the ability to visually discriminate between shapes, and we compared these gains across groups that trained with different regimens. In Experiment 1, we compared visual discrimination gains of participants who trained by actively tracing the shapes on a digital tablet using their non-dominant (left) hand with gains of non-active groups that trained by receiving various types of visual training. In Experiment 2, we compared motor parameters and visual discrimination gains of participants who trained by actively tracing the shapes with their non-dominant (left) hand versus participants who trained with their dominant (right) hand.

## Methods

We conducted two behavioral training experiments, both employing a between-subjects design. We evaluated the change in each participant’s ability to visually discriminate between shapes by conducting psychophysical assessments of visual discrimination between shapes before and after 2 days of training sessions, as well as after a week of retention. Each group underwent a distinct type of training.

### Participants

Sixty adults (age 19–35 years, mean = 24.15, SD = 3.1) participated in the two experiments. Experiment 1 (n = 40) included four training groups and Experiment 2 (n = 20) included two training groups. Each training group consisted of ten participants.

All participants were healthy, right-handed and had normal or corrected-to-normal vision. Hand dominance was determined by the Edinburgh handedness test (Oldfield, 1971), and only participants who met the criteria for right-hand dominance (a score between 61 and 100 and/or declaring that they always use the right and never use the left hand for both writing and drawing) were accepted to participate in the study.

### Stimuli

**Fig. 1:**
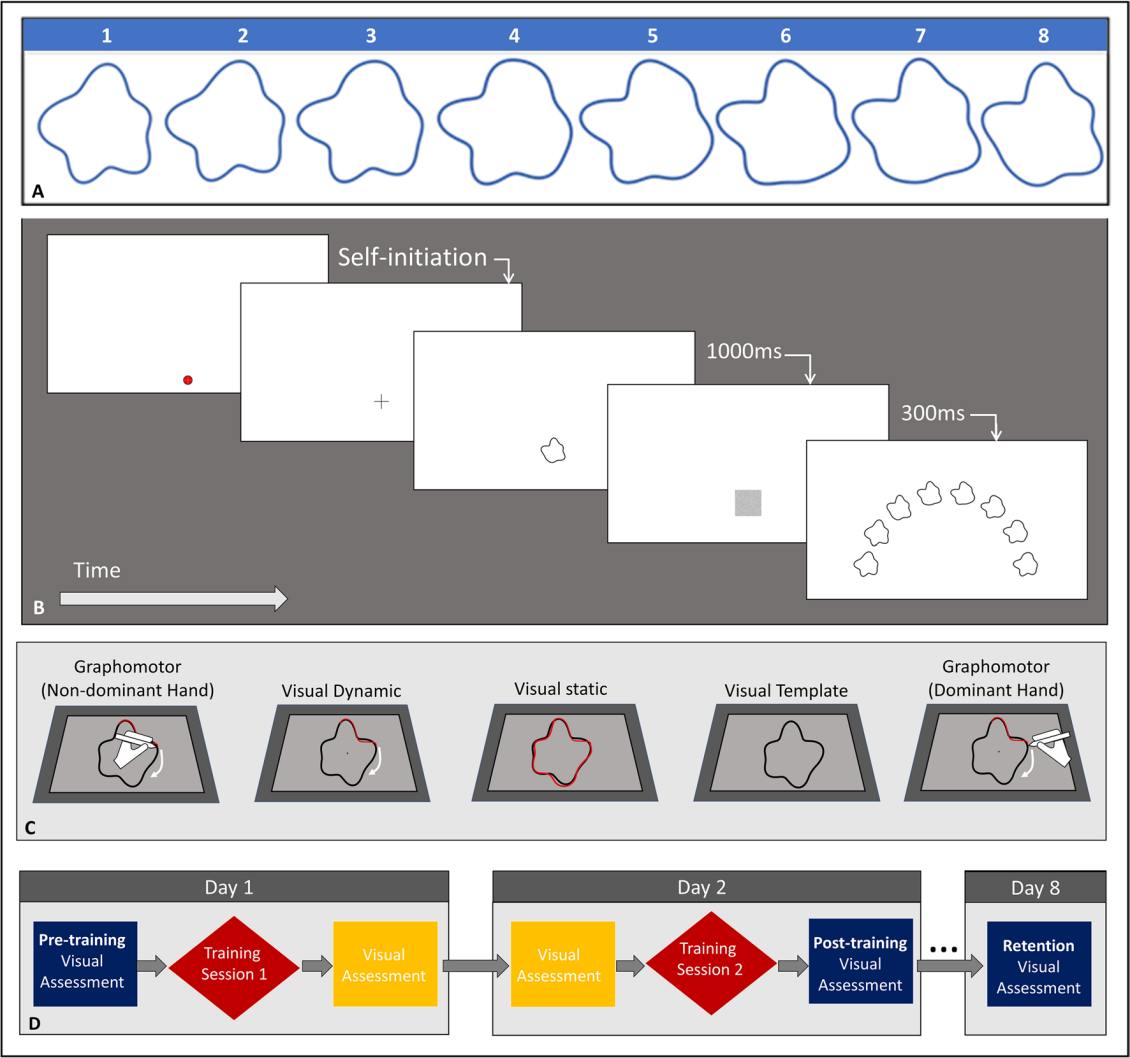
Experimental stimuli, tasks, and procedure. **(A)** Stimuli set. The set was constructed such that consecutive stimuli 1–8 were mathematically equidistant, while there were three steps between shapes 8 and 1 (see Online Supplemental Material for details). **(B)** Visual assessment consisted of a delayed match-to-sample task. Participants were presented with a target shape followed by a visual mask, and an array of all the eight shapes in the training set, from which they were asked to indicate which shape they think is identical to the target. **(C)** Training regimens. From left to right – Graphomotor Non-Dominant Hand (Experiments 1 and 2), followed by three visual training groups (Experiment 1), and Graphomotor Dominant Hand group (Experiment 2). **(D)** Experiment flow. Each participant completed 2 days of training sessions (according to the assigned type of training) and another visual assessment session 1 week later. Training sessions were preceded and followed by a visual assessment task. Red rhombuses represent the training phase. Rectangles represent the visual assessment phases: blue for the pre/post-training and retention assessments that were included in data analysis (Figs. 2 and 4) and yellow for the visual assessments not included in this analysis.

Stimuli (Fig. 1A) were closed contour amoeboid shapes, with protrusions and intrusions (‘bumps and dimples’). These types of shapes are considered ecologically relevant to visual characteristics of biological entities, as they can be easily modified to create natural shapes (e.g., faces; Wilson et al., 2002). We devised a novel stepwise stimuli synthesis technique to construct parametrically adjusted stimuli, so that the similarity between them is mathematically controlled and characterized. We empirically validated that our parameterization procedure corresponded with perceptual similarity (e.g., stimulus 1 is more confusable with stimulus 2 than with stimulus 4; see Fig. 1A for full stimuli set) by testing human visual discrimination between our generated stimuli (see Online Supplementary Material (OSM) for full details on stimuli synthesis and validation). A total of eight stimuli were generated, and the same set of stimuli was used across all training groups.

### Experimental setup

Throughout both experiments, we used a stylus and an upward-facing 21.5-in. Wacom DTU-2231 digitizing tablet, connected to a computer running MATLAB version 2021b (The MathWorks, Natick, MA, USA) with the Psychtoolbox-3 (Brainard, 1997; Kleiner et al., 2007) and the Repeated Measures packages (Friedman, 2014). The tablet was placed horizontally with a 25° tilt angle. It was adjusted to the height of each participant so that viewing distance was kept consistent at 40 cm.

### Visual assessment task

To assess participants’ ability to discriminate between similar shapes, we used a delayed match-to-sample task (Fig. 1B). Each trial was initiated by the participant tapping the designated location at the bottom center of the screen (marked as the home location, see red circle in Fig. 1B). The trial began with a fixation cross presented above this home location, followed by a presentation of the target shape for 1,000 ms, a 300-ms visual mask, and then a presentation of all the shapes in the sample set, organized in a semi-circle, equidistant from the home location. Participants were instructed to tap the sample shape that is identical to the target shape shown earlier. Each stimulus in the set served as a target eight times (yielding 8 × 8 = 64 trials, random presentation order). The location of samples along the semi-circle was counterbalanced across trials. Before the first visual assessment, participants were familiarized with the procedure using a different set of shapes.

### Training

*Experiment 1* included four training groups: (1) Graphomotor training with the non-dominant (left) hand, (2) Visual Dynamic, (3) Visual Static, and (4) Visual Template. *Experiment 2* included two training groups: (1) Graphomotor Non-Dominant hand, and (2) Graphomotor Dominant Hand (see Fig. 1C and detailed description below). In each experiment, participants were randomly assigned to one of the training groups.

*Graphomotor training* (Experiments 1 and 2) involved tracing the shapes on a digital tablet using an electronic stylus with full visibility of the hand and the stylus while receiving visual feedback of the trace on the tablet as it is formed (as if drawing with a pen on paper). In Experiment 1, tracing was conducted using participants’ non-dominant (left) hand, while in Experiment 2 participants were randomly assigned to tracing with either their dominant (right) or non-dominant (left) hand. In both studies, participants initiated each trial by pressing a key on a keyboard which triggered the appearance of one of the eight reference template shapes on the screen. Templates were traced continuously for one full cycle, at a comfortable natural pace within a time limit of 15 s per shape. The starting position and direction of tracing were freely chosen by the participant. Participants were instructed to start tracing as accurately and smoothly as possible immediately when the shape appeared, and to continuously trace until they returned to the starting point. Delays in starting to trace or stops in the tracing movement for longer than 400 ms resulted in automatically aborting the trial and notifying the participant. The order of shape presentation across trials was random. 

*Visual training *(Experiment 1) involved three types of observation training performed by three groups of participants. Each participant in the visual groups was randomly assigned to one reference participant from the non-dominant graphomotor group. Participants in the *Visual Dynamic* training group observed videos of traces (as they were originally collected from the yoked graphomotor training participant), overlaid on the corresponding reference template that was traced. Participants in the *Visual Static* training group observed static images of the full end-point trace produced by the yoked active participant, overlaid on the corresponding reference template. Participants in the *Visual Template *training group observed only images of the reference templates (without the visual trace that had been produced by the active participant). Duration of stimulus presentation in all Visual groups corresponded to the time it took the yoked active participant to trace the shape; thus, visual exposure time was kept consistent across training groups. The order of shape presentation across trials was also kept consistent between yoked participants.

For all training groups, shapes were presented in the center of the screen. The presentation size of the largest aspect of the shape was 4.9 cm, which from the viewing distance of 40 cm took up a visual angle of approximately 7°. The width of the reference template’s outline (black) and of the trace (red) was 3 mm. Each shape was traced or observed 25 times throughout each training session, for a total of 200 trials per training session.

*Familiarization and catch task.* To familiarize participants with the setup and verify compliance with task instructions, participants performed a pre-training block on day 1, in which they traced or observed shapes that were not included in the training set. To maintain and monitor participants' attention to the shape, 16 of the trials (two repetitions per shape, randomly interspersed) were catch trials. During catch trials, a certain segment of the template transiently (1,200 ms) changed in width (to 5 mm) while that specific segment was being traced. The participant’s task was to detect and report such changes at the end of each trial in response to a question (“Was there a change to the thickness of this shape during this trial? Yes/no”). Feedback regarding overall success on the catch task was given at the end of each day. All participants performed at ceiling on the catch task, and no datasets were discarded. Catch trials were kept consistent between yoked participants.

### Experimental procedure

Both experiments consisted of three sessions within an 8-day period: two sessions on days 1 and 2 for training, and another session on day 8 to measure retention of visual shape discrimination (Fig. 1D). Each of the two training sessions started with a pre-training assessment of visual discrimination, followed by a training phase (according to the assigned group), and ended with a re-assessment of visual discrimination. The third session included only a visual assessment task. Participants were informed that the purpose of the study was to explore improvements in their visual discrimination ability as a result of the training they undergo, and this was emphasized and reiterated before each of the visual assessments.

### Data analysis

#### Visual assessment outcome measures.

Visual discrimination performance was quantified by two measures, which were separately compared across groups and assessment time points within each experiment (for full details on how these were calculated, see the OSM).

1. *Accuracy.* Quantified as the proportion of correct matches (hits). Higher values correspond with better performance.
2. *Relative distance error.* Misses were quantified by accounting for the distance between the target stimulus and the chosen match. Due to the procedure by which the stimuli were constructed, confusing stimulus 1 with stimulus 2 corresponds with a smaller perceptual error relative to confusing it with stimulus 5 (although both would be counted as a mistrial in the accuracy measure). As an error measure, lower values correspond with better performance.

#### Tracing outcome measures.

We calculated several measures to assess tracing performance of the graphomotor groups: duration, accuracy, and smoothness (see Fig. 3A for an illustration). The tracing measures were calculated for all valid non-catch trials. The evolution of each measure with training was examined by comparing the mean value of each measure between the two training sessions (on day 1 and day 2).

1. *Tracing duration* (seconds). Tracing time of each shape, measured from the first contact of the stylus with the tablet until trial completion (movement stop or stylus lift).
2. *Tracing accuracy* (cm^2^). Overall difference between the template and its trace, measured as the area enclosed between them (Unell et al., 2021). Traces that are superimposed on the reference template exactly (high spatial accuracy) will have zero area between them reflecting higher accuracy, whereas imperfect tracing (lower spatial accuracy) will lead to a larger area.
3. *Smoothness of tracing movement* (dimensionless). A kinematic characteristic of the dynamic evolution of tracing, quantifying intermittent changes between acceleration and deceleration, thus reflecting the fluctuations in the speed of the tracing movement across time. Smoothness is often used as a measure of the quality of a movement. We measured movement smoothness by an index (spectral arc length, SPARC; Balasubramanian et al., 2015), which quantifies complexity of the frequency content in the movement. A higher SPARC indicates less high-frequency components – a reflection of increased movement smoothness and better performance.

## Results

## Experiment 1: Effects of non-dominant (left) hand graphomotor engagement on visual learning

The aim of this experiment was to compare the effect of training with graphomotor engagement of the non-dominant hand vs. passive visual training (Visual Dynamic, Visual Static, Visual Template) on visual discrimination performance. To this end, we analyzed two measures of visual discrimination (accuracy and distance error) at three time points – before training, immediately after day 2 of training, and 1 week after training.

### Pre-training group comparison

Before assessing the effects of training, we first examined whether there were any differences between the groups prior to training. This was accomplished by performing a one-way ANOVA on the pre-training performance measures (accuracy and distance error) on data from the four training groups (including Graphomotor Non-Dominant Hand, Visual Dynamic, Visual Static, and Visual Template training groups). We did not find a group difference in either pre-training accuracy (F(3,36) = 1.5, p = 0.23, η^2^_p_ = 0.11) or distance error (F(3,36) = 0.59, p = 0.62, *η*^*2*^_*p*_ = 0.05).

### Visual discrimination performance analysis

**Fig. 2:**
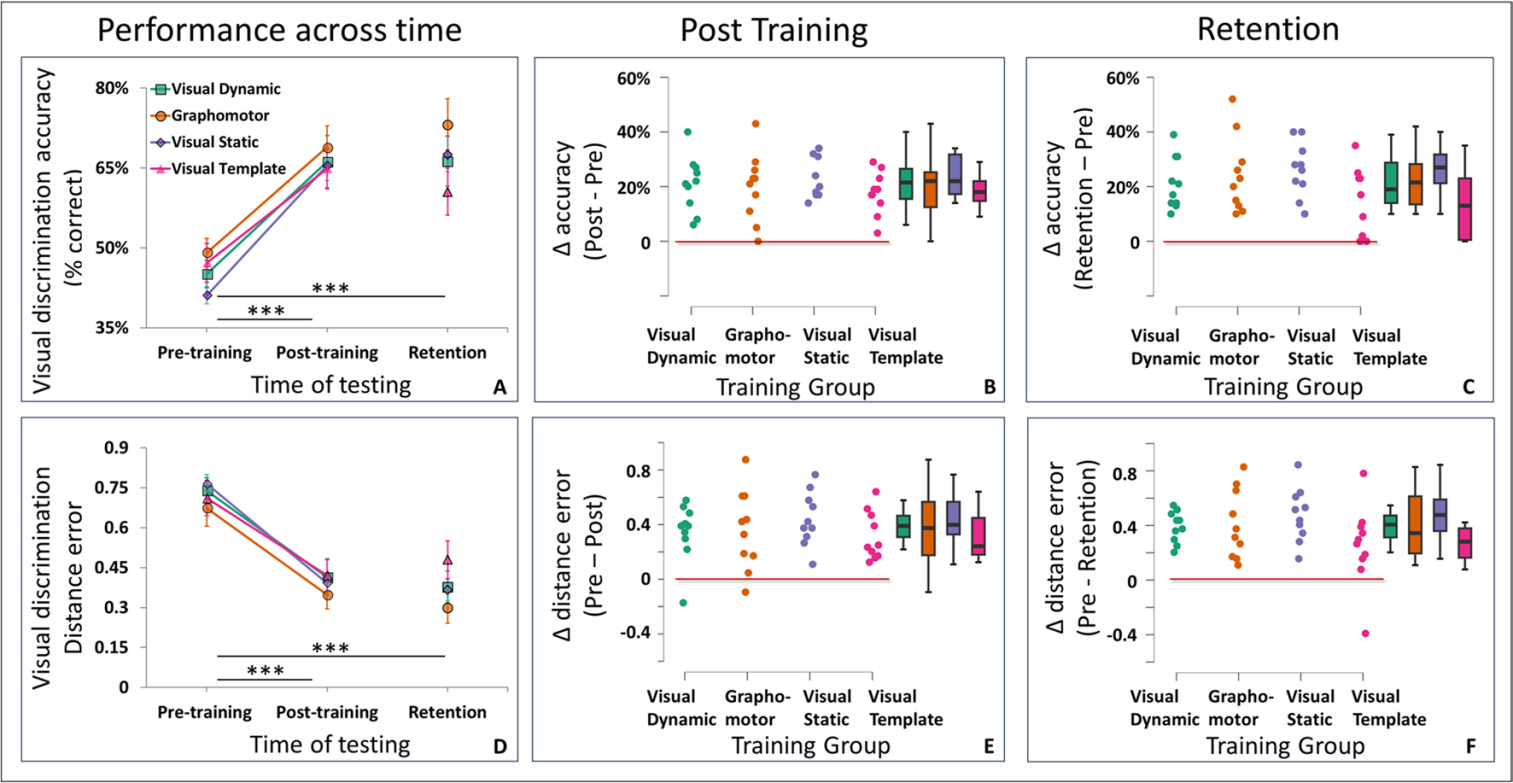
Visual learning from* non-dominant hand graphomotor* and *visual* training regimens. **(A)** Accuracy (group data). Group means ± SEM, significance of post hoc differences denoted by three asterisks (***) for p < .001. Visual discrimination accuracy significantly improved after training, and these gains persisted 1 week later. **(B and C)** Baseline adjusted measures. Individual participant changes in accuracy (Δ, after subtracting baseline performance from performance after training and from performance a week later). The line at zero corresponds with no learning. **(D–F)** The same format as A–C, presented for the distance error measure. Lower error values in D correspond with better performance.

To assess visual discrimination performance across the three time points (before training, after training, and 1 week after training completion) we performed a mixed design ANOVA, with Time as a within-participant factor and Group as a between-participant factor. We conducted this analysis separately for the accuracy (Fig. 2A) and distance error (Fig. 2D) measures.

#### *Accuracy (Fig.* *2**A)*

##### Improvement across time.

The analysis of visual discrimination accuracy revealed a significant increase in visual discrimination accuracy after training, and a persistence of this gain in performance 1 week later. This was indicated by significant effects of Time on performance (F(1.7, 61.9) = 117.6, p < 0.001, *η*^*2*^_*p*_ = 0.77; Greenhouse–Geisser corrected due to violation of sphericity indicated by Mauchly’s test showing a significant difference in variation between the groups (W(2) = 0.83, p = 0.04; mean ± SD of accuracy: pre-training 45.63% ± 8.30%, post-training 66.38% ± 12.33%, retention 66.93% ± 13.6%). Post hoc testing with the Bonferroni correction indicated a significant increase in accuracy between pre-training and post-training sessions (mean increase of 20.7%, SD = 9.6%, p < 0.001, d = -1.76), with this improvement maintained at the retention phase (mean increase between pre-training and retention of 21.2%, SD = 12.2%, p < 0.001, d = -1.80). Note that after training, discrimination accuracy was not at ceiling for any participant, but ranged between 45% and 92% across participants. No significant difference was observed between post-training and retention performance (mean difference 0.6%, SD = 8.4%, p = 1, d = -0.05).

##### Difference between groups.

No significant differences were found between the training groups in terms of accuracy. This was reflected by the insignificant main effect of Group (F(3,36) = 0.74, p = 0.53, *η*^*2*^_*p*_ = 0.06; mean ± SD of accuracy: Graphomotor Non-Dominant Hand 63.73% ± 12.11%, Visual Dynamic 59.2% ± 12.87%, Visual Static 58.17% ± 8.3%, Visual Template 57.57% ± 12.41%), and insignificant interaction between Time and Group (F(5.2,61.9) = 1.7, p = 0.14, *η*^*2*^_*p*_ = 0.13).

##### Bayesian analysis.

To further quantify the findings from null hypothesis significance testing, we conducted a Bayesian mixed-design ANOVA, with Time as a within-participants factor and training Group as a between-participants factor. This analysis revealed that the data were best represented by a model that includes only the Time factor. The Bayes factor BF_01_ for the Time model was 2.66*10^–21^, indicating decisive evidence in favor of this model when compared to the null model, thus further supporting the significant effect of Time on accuracy increase that was found by null hypothesis significance testing. When comparing the effect of Group to the null model, the Bayes factor indicated a preference for the null model (BF_01_ = 6.06), suggesting further support that the amount of improvement in discrimination accuracy was not affected by the type of training regimen. Finally, the Bayes factor BF_01_ for the Time × Group interaction model was 1.71, indicating anecdotal evidence in favor of a null interaction.

#### *Distance error (Fig.* *2**D)*

##### Improvement across time.

The analysis of distance error revealed a significant decrease in error after training, and a persistence of this error reduction 1 week later. This was evident from the significant effect of Time on distance error (F(1.7,61,9) = 116.6, p < 0.001, *η*^*2*^_*p*_ = 0.76; Greenhouse–Geisser corrected due to violation of sphericity; mean ± SD of distance error: pre-training 0.72 ± 0.17, post-training 0.39 ± 0.17, retention 0.38 ± 018). Post hoc testing with Bonferroni correction indicated a significant decrease in distance error between pre-training and post-training sessions (mean reduction of 0.33, SD = 0.03, p < 0.001, d = 1.83) with this reduction maintained at the retention phase (mean reduction between pre-training and retention of 0.34, SD = 0.03, p < 0.001, d = 1.9). Distance error after training ranged between 0.03 and 1.15 across participants. We did not observe a significant difference in distance error between post-training and retention (mean difference 0.01, SD = 0.03, p = 1, d = 0.08).

##### Difference between groups.

No significant difference was found between the training groups in terms of distance error, as reflected by the insignificant main effect of Group (F(3,36) = 0.73, p = 0.54, η^2^_p_ = 0.06; mean ± SD of distance error: Graphomotor Non-Dominant Hand 0.46 ± 0.21, Visual Dynamic 0.54 ± 0.22, Visual Static 0.55 ± 0.15, Visual Template 0.58 ± 0.26), and insignificant interaction between Time and Group (F(5.2,61.9) = 1.20, p = 0.32, *η*^*2*^_*p*_ = 0.09).

##### Bayesian analysis

To further quantify the findings from null hypothesis significance testing, we performed a Bayesian mixed design ANOVA, which revealed that the data were best represented by a model that includes only the Time factor (BF_01_ was 6.84*10^–22^), indicating decisive evidence in favor of the effect of Time compared to the null model. When comparing the effect of Group to the null model, the Bayes factor indicates a preference for the null model (BF_01_ = 6.63), providing further support that the amount of reduction in distance error was not affected by the type of training regimen. Bayes factor BF_01_ for the Time × Group interaction model was 4.07, indicating moderate evidence in favor of the model of a null interaction.

*Taken together*, the accuracy and distance error results supported a general improvement in visual discrimination performance across time, with no discernible difference between graphomotor and any of the visual training groups. The Bayesian analysis suggested that an interaction between Time and Group could not be ruled out, indicating a possibility that the visual discrimination performance was differently modulated across time by different types of training (graphomotor or visual). Next, this possibility was further examined by separately testing post-training and retention performance, adjusted by each individual’s baseline performance (see below).

### Baseline-adjusted measures

Although the visual discrimination performance analysis described above did not reveal significant group differences in baseline performance (measured prior to training, see above), individual differences still exist and may mask potential group effects at later time points. To address this issue, we also examined group differences after subtracting for each participant their corresponding baseline performance, both post-training (Figs. 2B, 2E) and at retention testing (Figs. 2C, 2F). A separate one-way ANOVA for each time point (post-training/retention) and measure (accuracy/distance error) was performed with the adjusted measure (ΔAccuracy/ΔDistance error) serving as the dependent variable, and the four training groups as fixed factors.

#### *Baseline-adjusted accuracy (Figs.**2**B, 2C)*

With respect to Δaccuracy, the analysis of *post-training* data did not reveal a difference between training groups (F(3,36) = 0.75, p = 0.52, η^2^_p_ = 0.06; mean ± SD of post-training Δaccuracy: Graphomotor Non-Dominant Hand 19.8% ± 12.38%, Visual Dynamic 21.1% ± 10.04%, Visual Static 24.1% ± 7.91%, Visual Template 17.7% ± 7.83%). Bayesian analysis further supported this by showing moderate evidence in favor of the null hypothesis (BF_01_ = 3.94). Similar analysis on the *retention* data did not reveal a significant effect of Group either (F(3,36) = 2.29, *η*^*2*^_*p*_ = 0.161, p = 0.09; mean ± SD of retention ΔAccuracy: Graphomotor Non-Dominant Hand 24.1% ± 13.83%, Visual Dynamic 21.2% ± 9.57%, Visual Static 26.2% ± 9.94%, Visual Template 13.4% ± 12.85%), but the evidence from Bayesian analysis supporting null group differences in retention was only anecdotal (BF_01_ = 1.4).

#### *Baseline-adjusted distance error (Figs.**2**E, 2F)*

With respect to ΔDistance error, the *post-training* data analysis revealed no difference between groups (F(3,36) = 0.57, p = 0.64, η^2^_p_ = 0.05; mean ± SD of Training ΔDistance error: Graphomotor Non-Dominant Hand 0.36 ± 0.29, Visual Dynamic 0.35 ± 0.21, Visual Static 0.44 ± 0.2, Visual Template 0.32 ± 0.18), and Bayesian one-way ANOVA indicated moderate evidence in favor of the null model (BF_01_ = 5.55). We also did not find a significant effect of Group in *retention* (F(3,36) = 1.7, p = 0.2, η^2^_p_ = 0.13; mean ± SD of Retention ΔDistance error: Graphomotor Non-Dominant Hand 0.41 ± 0.25, Visual Dynamic 0.39 ± 0.11, Visual Static 0.48 ± 0.2, Visual Template 0.25 ± 0.3), but the evidence from Bayesian analysis supporting null group differences was only anecdotal (BF_01_ = 1.88).

*In summary of Experiment 1,* all training groups similarly improved across time, and we did not detect an advantage for graphomotor over visual training in the amount of improvement after training. Similarly, we did not detect significant group differences in maintaining improvements at retention testing, although this result is less robust based on the Bayesian analysis, which was less conclusive regarding a group difference in the persistence of increased accuracy and decreased distance error a week after training completion.

## Experiment 2: Effects of dominant versus non-dominant hand graphomotor engagement on visual learning

The lack of difference in visual discrimination performance between the non-dominant hand graphomotor training group and the visual training groups suggests that motor pathways controlling the non-dominant hand do not have a facilitating effect on visual discrimination of shapes above and beyond passive visual training. To determine whether this is the general case for motor pathways, or an effect unique to the specific motor pathways controlling the non-dominant hand, we performed another experiment in which we compared visual discrimination between dominant and non-dominant hand graphomotor training groups. To this end, we collected data from a new group of 10 participants who trained with their non-dominant (left) hand, and a group of 10 participants who underwent graphomotor training using their dominant (right) hand.

### Comparison of tracing skills between dominant and non-dominant hand

To compare movement parameters of the two groups (Dominant vs. Non-Dominant Hand) across the two training sessions, we performed a mixed design ANOVA for each of the movement parameters. The tested parameter (duration, accuracy, or temporal smoothness) across Time (first and second day) served as a within-participant factor, and Hand (Dominant vs. Non-Dominant Hand training groups) as a between-participant factor.

**Fig. 3:**
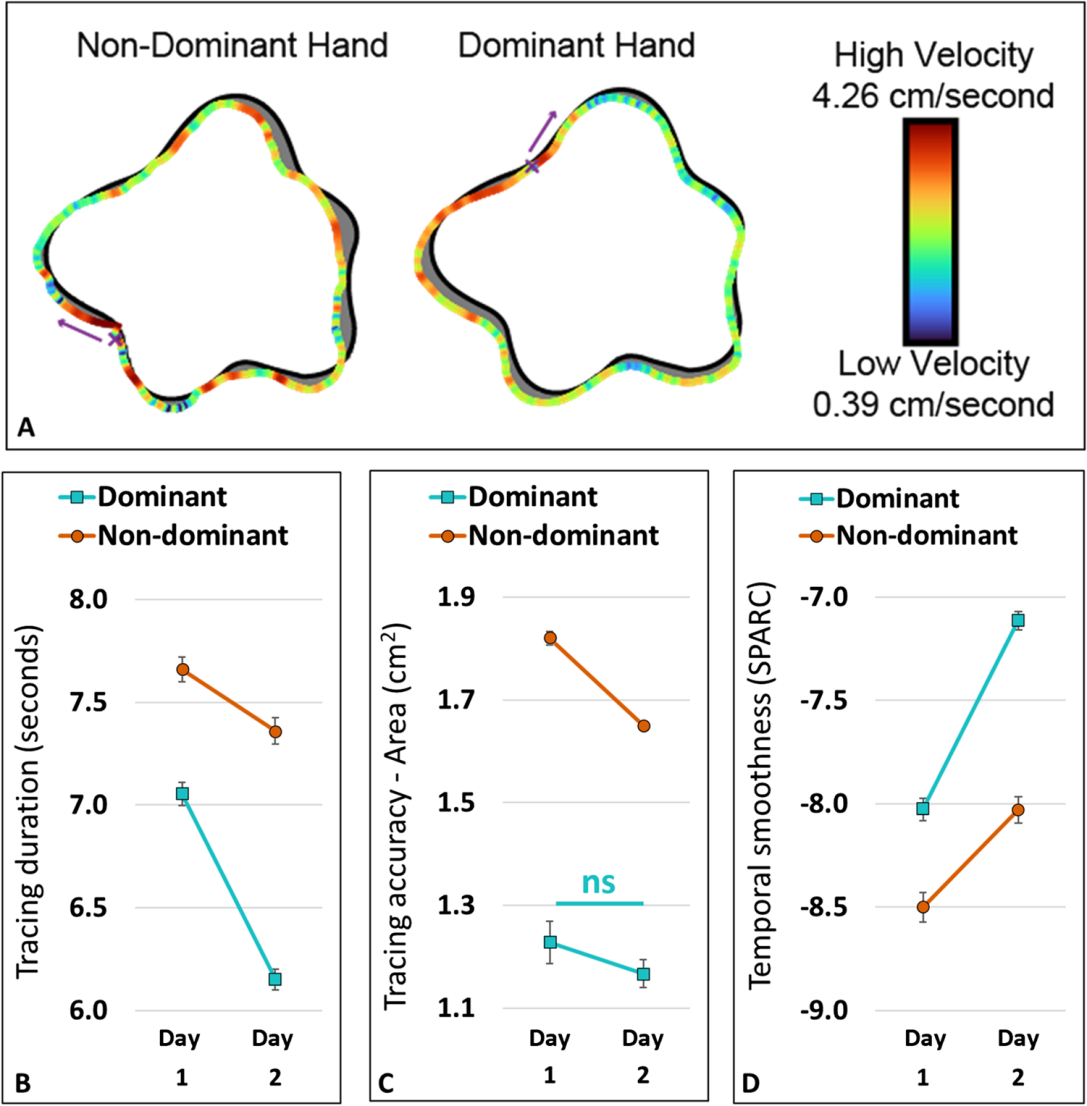
Tracing skills of Graphomotor groups (Dominant and Non-Dominant Hand) across time. **(A)** An example trace of a participant from the Dominant Hand Graphomotor training group (right), and of a participant from the Non-Dominant Hand Graphomotor group (left). The template is shown in black and the produced trace is color-coded for velocity. The tracing starting point is marked by an X and the direction of tracing by an arrow. The gray area between the trace and template represents the tracing error (area between the template and its trace, used to quantify tracing accuracy). **(B–D)** Motor parameters comparison between hands, across sessions. Group means ± SEM are shown. Significance of post hoc differences between the training groups and across training days were all p < .001, excluding Dominant Hand group across days in the tracing accuracy area measure (marked *ns*). On average, the dominant hand was **(B)** quicker (shorter tracing duration), **(C)** more accurate (smaller area between template and trace), and **(D)** more temporally smooth (higher SPARC value) than the non-dominant hand. Across sessions, the dominant hand improved more than the non-dominant hand on tracing duration and the temporal smoothness of the tracing movement. However, only the non-dominant hand improved in tracing accuracy.

We found that measures of tracing duration, tracing accuracy, and temporal smoothness all significantly differed between the Dominant and Non-dominant Hand groups (main effect of Hand in all three comparisons as well as all Bonferroni-corrected comparisons between hands for all parameters p < 0.001). See Fig. 3A for an illustration of one trial and Fig. 3B-D for group results. All parameters showed an interaction between Hand and Time (p < 0.001) and differed between the two sessions (main effect of Time for all these measures, p < 0.001), with the Dominant Hand showing more between-session improvement in tracing duration (Fig. 3B) and temporal smoothness (Fig. 3D), but only the Non-Dominant Hand showing improvement in tracing accuracy (Fig. 3C). The Dominant Hand group did not show significant improvement in tracing accuracy (p = 0.55) between the two training sessions but became significantly quicker and smoother (p < 0.001) across time, whereas the Non-Dominant Hand group improved on all measures (all p < 0.001).

### Comparison of visual discrimination between dominant and non-dominant hand graphomotor training groups

### Pre-training group comparison

**Fig. 4:**
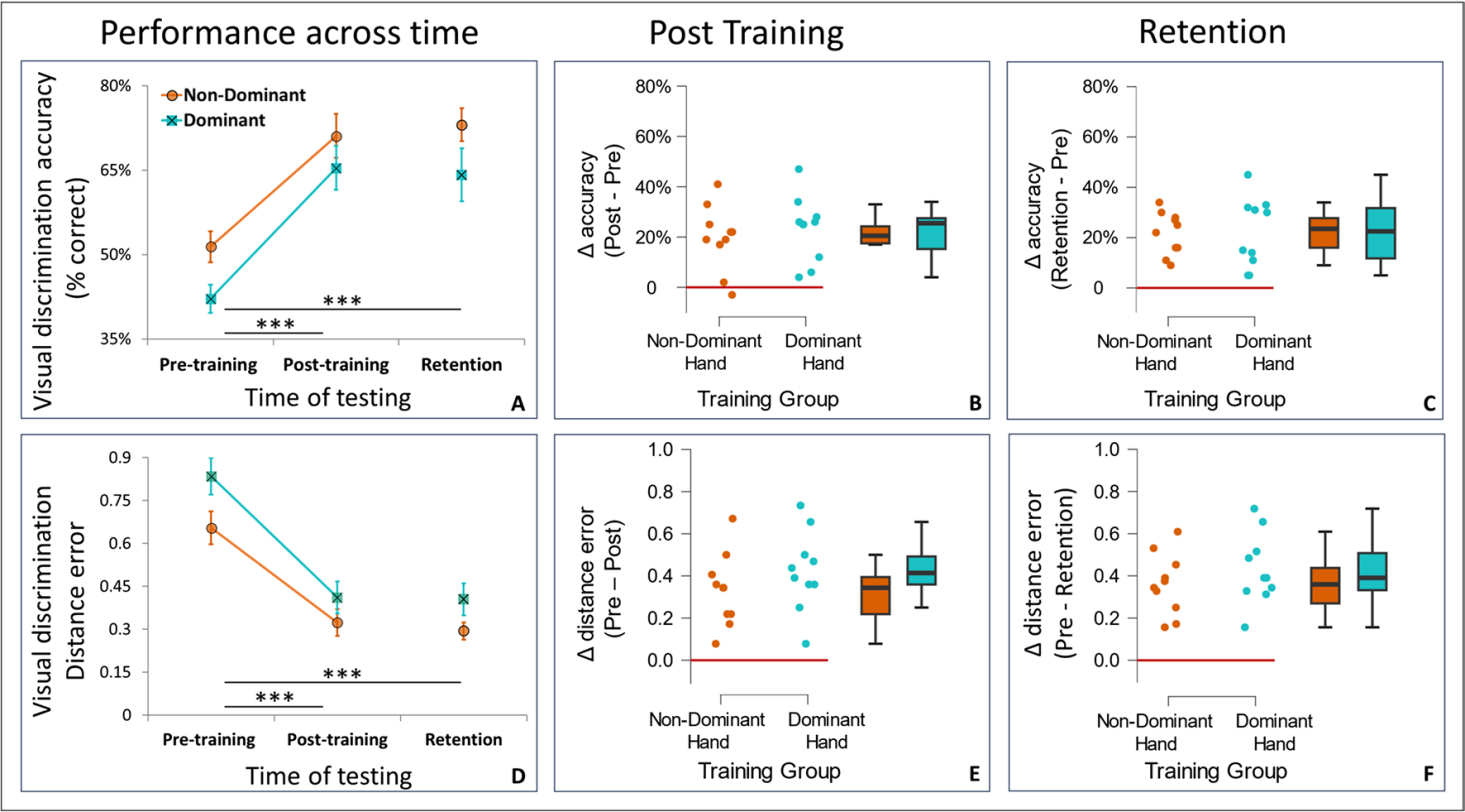
Visual learning from *graphomotor* training across the *non-dominant* and *dominant *hand training groups. (**A**) Group means ± SEM, significance of post hoc differences denoted by three asterisks (***) for p < .001. Both hand groups improved in visual discrimination performance following training, but differences between the hands did not reach significance. **(B** and **C)** Baseline adjusted measures. Individual participant accuracy (Δ after subtracting baseline performance from performance after training and from performance a week later). The line at Zero corresponds with no learning. **(D–F)** The same format as A–C for the distance error measure. Lower error values in D correspond with better performance.

We first examined whether there were any differences between the groups prior to training. This was accomplished by performing a Student’s t-test comparing pre-training performance measures (accuracy and distance error) between the two training groups (Graphomotor Non-Dominant Hand, Graphomotor Dominant Hand). We found a significant advantage in visual discrimination accuracy for the Non-Dominant Hand training group before training (t(18) = 2.5, p = 0.02, d = 0.51; Non-Dominant Hand mean = 51.4%, SD = 8.9%; Dominant Hand mean = 42.1%, SD = 7.8%) and a near significant advantage in distance error before training (t(18) = 2.09, p = 0.05, d = 0.5; Non-Dominant Hand mean = 0.65, SD = 0.18, Dominant Hand mean = 0.84, SD = 0.20). This indicates a baseline advantage in visual discrimination performance for the non-dominant hand group over the dominant hand group. Considering these differences in baseline performance between the two groups, the most informative analyses of group effects are baseline-adjusted measures of individual subjects (see below), which are not affected by these pre-training group differences. For non-adjusted measures of visual discrimination performance across time, see Figs. 4A and 4D, and for corresponding statistical analysis see OSM.

### Baseline-adjusted measures

Considering the group differences in visual discrimination at baseline (see above), we examined group differences at post-training and retention after subtracting the corresponding baseline measure of individual subjects. Separate t-tests for each time point (post-training/retention) and measure (accuracy/distance error) were performed on these baseline-adjusted measures (Figs. 4B, 4C and 4E, 4F).

#### *Baseline-adjusted accuracy (Figs.**4**B, 4C)*

With respect to ΔAccuracy, the *post-training* analysis did not reveal a difference between groups (t(18) = -0.62, p = 0.54, d = -0.28; mean ± SD of ΔAccuracy Post Training: Graphomotor Non-Dominant 19.7% ± 12.94%, Graphomotor Dominant 23.3% ± 13%). A Bayesian analysis further supported this by showing only anecdotal evidence in favor of the null hypothesis (BF_01_ = 2.2). Similar analysis on ΔAccuracy in *retention* was run with a two-sample unequal variance Student’s t-test due to the Brown-Forsythe test suggesting a violation of the equal variance assumption (F(1,18) = 5.53, p = 0.03). ΔAccuracy in Retention data did not reveal a significant effect of Group either (t(18) = -0.06, p = 0.95, d = -0.03; mean ± SD of ΔAccuracy of Retention: Graphomotor Non-Dominant 21.8% ± 8.43%, Graphomotor Dominant 22.1% ± 13.77%). The evidence from Bayesian ANOVA supporting null group differences in Retention was only anecdotal (BF_01_ = 2.51).

#### *Baseline-adjusted distance error (Figs.**4**E, 4F)*

With respect to ΔDistance error, the *post-training* data did not reveal a difference between groups (t(18) = -0.62, p = 0.54, d = -0.28; mean ± SD of ΔDistance error of Post-Training data: Graphomotor Non-dominant 0.33 ± 0.17, Graphomotor Dominant 0.42 ± 0.19). A Bayesian t-test further supported this by showing only anecdotal evidence in favor of the null hypothesis of no difference between groups (BF_01_ = 2.2). We also did not find a significant effect of Group in *retention* data (t(18) = -0.06, p = 0.95, d = -0.03; mean ± SD of ΔDistance error in Retention: Graphomotor Non-Dominant 0.36 ± 0.15, Graphomotor Dominant 0.43 ± 0.17). The Bayesian t-test showed that evidence in support of the null group differences was only anecdotal (BF_01_ = 1.8).

*In summary of Experiment 2,* motor and visual learning showed no evidence of dependence. A dominant-hand advantage was evident in all tracing motor parameters, as well as a difference between the hands in the amount of improvement in tracing motor parameters between the sessions. Despite this very prominent difference between the motor output of the two hands and its progression across time, we did not find a corresponding prominent difference in the degree of visual learning.

## Discussion

In the current study we probed the effect of motor engagement with the non-dominant (left) hand on visual perception of shapes. While participants improved on the visual discrimination task following graphomotor training, to our surprise these performance gains were not significantly different from gains obtained following visual (non-motor) forms of training (Experiment 1), or graphomotor engagement with the dominant (right) hand (Experiment 2) despite significant motor differences between the hands. These results are not likely to be explained by ceiling effects, as none of the participants achieved perfect performance. This similarity in visual discrimination performance gains after training is further supported by the Bayesian analysis, although potential differences between groups in the retention of improvements cannot be ruled out based on the strength of the evidence.

Thus, our results suggest that motor performance was not the critical factor underlying improvement on the visual discrimination task, and that specific parameters of the motor commands did not contribute to visual discrimination beyond the contribution of visual information. In fact, observation of the shape templates was sufficient to improve visual discrimination, as the group of participants who observed only templates reached similar performance gains as the graphomotor training group. Thus, our findings suggest that the training’s visual component was adequate for driving training-induced improvements in visual discrimination.

Our results are in contrast with an advantage of graphomotor training with the dominant hand for visual grapheme discrimination and recognition, which has been described in the literature about handwriting. A recent meta-analysis (Araújo et al., 2022) that included 50 studies examining this effect concluded that graphomotor training can induce a moderate to large effect on visual learning of graphemes, usually excelling over other types of non-motor training. However, teasing apart the contribution of different components of graphomotor activity (Araújo et al., 2022; Wiley & Rapp, 2021) revealed that while there is a large advantage of graphomotor training over non-graphomotor training (e.g., training via typing graphemes), its advantage over visual-only training (passive visual exposure) is smaller. This suggests that a substantial part of the advantage of graphomotor activity may be attributed to the visual exposure component, which is in agreement with the findings of the current study. Additionally, although most studies in the field have shown some advantage of graphomotor over visual training, other studies using stimuli with well-controlled differences (Koenigsberg, 1973; Naka & Naoi, 1995; Zhai & Fischer-Baum, 2019) report comparable effects of graphomotor and visual training on visual learning. For example, the recognition of Chinese characters and pseudo-characters did not differ after learning through practice that included visual learning, with or without handwriting (Feng et al., 2022). In another example, gains in visual recognition of letter-like synthesized symbols were similar following composition (active assembling of a symbol by selecting its elementary visual features) versus handwriting (Seyll et al., 2020).

## Support for a visual analysis mechanism underlying visual learning

Two opposing perspectives have been proposed for explaining the mechanisms that underlie the documented facilitation of visual learning by graphomotor engagement. One perspective (*embodied cognition*; Glenberg et al., 2013; Wilson, 2002) posits that sensorimotor interactions are critical for the advantage of graphomotor engagement in visual learning of graphemes. According to this perspective, the association between motor and sensory signals is built through the detailed motor reproduction of the graphemes’ shape and its coupling with highly predictable visual feedback that accompanies it (James & Atwood, 2009). This is thought to establish and strengthen neural functional pathways between the visual and motor systems, resulting in a “distributed functional neural network” serving both handwriting and reading activities (James & Gauthier, 2006; Longcamp et al., 2016). Thus, the motor programs and sensorimotor representations of trained graphemes can be reactivated or simulated when the need to recognize them and discriminate them from other graphemes arises (Labat et al., 2020; Xu et al., 2020). Motor programs have been shown to be useful for accessing knowledge of the visual form of letters and words even for a pure alexic patient, who showed improved mental imagery of graphemes after finger tracing them (Bartolomeo et al., 2002). The second perspective (*symbolic, abstractionist*; Goldinger et al., 2016; Mahon & Hickok, 2016) posits that graphomotor engagement per se is not the crucial component driving the learning advantage attained by graphomotor training. Rather, perceptual processes that are associated with graphomotor activity are the ones responsible for visual learning. Accordingly, any training that encourages relevant exposure and enhances attention to the critical characteristics of a visual stimuli will encourage feature-based visual processing. This will result in efficient visual graph recognition (Gibson et al., 1962; Grainger, 2018; Pelli et al., 2006) and a similar benefit as graphomotor experience, irrespective of the motor act. In the current study, we found evidence in support of the symbolic perspective. Although graphomotor training induced sensorimotor associations, it did not provide an added benefit for visual learning over visual-only training, implying that embodiment is not obligatory for visual learning of shapes through tracing.

A mechanism which is proposed to explain the added value of the perceptual processes associated with graphomotor engagement suggests that it is caused by exposure to variable exemplars, as created during repeated production (*perceptual variability mechanism*; James & Engelhardt, 2012; Li & James, 2016; Vinci-Booher & James, 2020). In the current study, we did not find evidence in support of this mechanism, since there was no visual learning advantage for non-dominant hand tracing, despite a difference in accuracy between the tracing output of the dominant versus non-dominant hands (i.e., the dominant hand producing more accurate, and thus less variable traces than the non-dominant hand). This suggests that the increased perceptual variability provided by non-skilled tracing did not contribute to visual learning. A similar conclusion can be drawn from the lack of added benefit for visual learning from presentation of the visual trace on top of the templates (Visual Static and Visual Dynamic trainings), versus observation of the templates alone (Visual Template training). That is, the addition of the visual trace provides variable visual exemplars of each shape but does not impact visual learning further than the improvement induced by observing the templates. An important caveat is that we have not directly compared the amount of visual variability presented by the output of non-dominant-hand tracing (in the current study) with visual variability that may result from free-form writing/copying. This is important since previous work arguing for perceptual variability as a driver for the beneficial outcomes of learning through writing has found that such effects are not replicated with tracing (James & Engelhardt, 2012; Li & James, 2016). To the best of our knowledge, our study is the first, to examine the effect of visual variability produced by an unskilled hand on visual discrimination learning. Employing quantification of motor output in future studies, such as the spatial accuracy measure implemented in the current study, would allow comparisons between different sources for visual variability in different studies, and help characterize the dimensions of visual variability which may contribute to visual learning.

The fact that training with the visual template alone was sufficient for fostering visual learning (i.e., induced the same magnitude of visual learning as all other groups) suggests that the process that supported this visual learning was perceptual in nature. This result is in line with the *visual analysis mechanism* (Fernandes & Araújo, 2021; Kaufman, 1980; Seyll et al., 2020), which proposes that visual learning depends on increased awareness of the critical distinctive features of graphemes and on enhanced visual processing of their diagnostic visual features (Nelson & Wein, 1974; Pick, 1965; Samuels, 1973; Tawney, 1972). This can be achieved via the active process of consecutively producing a grapheme, but can be similarly achieved with non-motor regimens in which the parameters of the training encourage the participant to focus on the grapheme’s relevant visual features (the same ones which drive the advantage of visual learning through graphomotor activity under other circumstances, e.g., segmentation of the grapheme into subcomponents; Courrieu & de Falco, 1989; Seyll et al., 2020). One such parameter for encouraging focus on relevant visual features is the granularity of differences between the graphemes in the learning set. It has been proposed that the fine-grained differences between stimuli force the participant’s attention to the distinctive features of highly confusable pairs (Araújo et al., 2022; Samuels, 1973; Tawney, 1972). The stimuli-set of the current study was designed and validated as presenting very fine-grained differences between shapes, with the distinguishing features parametrically adjusted for stepwise control over their visual similarity (see OSM for details on stimuli construction and validation). This emphasis on fine-grained differences may have increased the likelihood that the attention of the observer was sufficiently drawn to the distinguishing features of shapes through visual inspection alone.

## Object-like shapes versus graphemes

The divergence of our results from previous studies reporting an advantage of handwriting for visual learning may be connected to the different types of stimuli used. While most prior studies tested graphemes, our study focused on object-like shapes. The structural and conceptual differences between these stimulus types suggest that visual learning of object-like shapes may be mediated differently than learning of graphemes. Consequently, graphomotor experience might not impact the learning of these stimulus types in the same manner. An intriguing possibility is that the natural development of writing systems, including the selection of graphemes with specific structures, could be influenced by how easily they can be learned through handwriting practice.

One structural difference is that in the current study, each stimulus was produced with one long curved stroke, whereas graphemes are typically composed of several strokes, each motorically corresponding to a portion of movement between two direction changes or pen lifts. Another structural feature that is absent from our stimuli but typical of graphemes, is sharp angles, which require pauses and punctate changes in movement direction for their production. Such edges, angles, and terminations are not only emphasized by the motor system as boundaries of movements but are also easily detectable by the visual system, which processes their orientation faster and more efficiently than curved lines (Wilson & Wilkinson, 2015). Relying on such features that are emphasized by both the motor and visual systems for mapping between motor and visual parameters is an efficient approach which is applied by learners (Smith et al., 2014). Indeed, recognition of graphemes has been shown to specifically rely on vertices (Lafontaine & Kolinsky, 2019). However, our specific stimuli, which lack these features, may not offer a learning advantage for graphomotor production.

A second structural difference is related to the visual feature of contour closure. While object-like shapes such as our stimuli are completely enclosed by a continues line which defines their boundary, graphemes can contain open as well as closed contours. This distinction is functionally relevant given the fundamental role of closed contours in organizing visual scenes into distinct objects (Moore et al., 1998), and the behavioral advantage in visual processing of closed versus open contours (including visual discrimination, recognition and detection; Elder & Zucker, 1993; Garrigan, 2012; Kovács & Julesz, 1993; Mathes & Fahle, 2007; Saarinen & Levi, 1999). Neurophysiologically, closure has been shown to induce neural activity in the lateral occipital complex (LOC; Altmann et al., 2003; Doniger et al., 2000; Sehatpour et al., 2008), a region that specializes in object perception (Grill-Spector et al., 2001). In contrast, grapheme recognition is underpinned by the left ventral occipitotemporal cortex (vOT; Dehaene et al., 2005; Rothlein & Rapp, 2014).

A conceptual difference is that graphemes are symbolic entities that carry linguistic meaning and are associated with sounds and a name, facilitating the integration of orthographic knowledge with visual processing (Lally & Rastle, 2023), whereas our object-like stimuli lacked these associations. Although the participants in our study all spontaneously named the shapes during the training phase, this was not part of their task, whereas in some of the previous studies, naming or familiarization with letters and the sounds they represent were part of the experimental protocol, akin to the process of acquiring literacy (e.g., Li & James, 2016). These conceptual distinctions may further contribute to the differing impacts of graphomotor experience on visual learning between the two types of stimuli.

## Limitations

Although subjects were randomly allocated to groups, in Experiment 2 we found differences in baseline visual discrimination performance between the groups. We addressed this issue by adjusting the individual post training and retention performance by the corresponding baseline performance. Still, the baseline group differences might have introduced the near-significant group effect in the Time × Group ANOVA. Future studies might consider pre-allocation of participants according to baseline performance.

While the evidence for lack of group difference post training was robust, the Bayes evidence supporting null differences between groups in retention was only anecdotal. Thus, we cannot rule out that benefits of graphomotor experience are manifested in retention. Nevertheless, evidence from the current study suggests that such effects, if they exist, are not strong and might require larger group sizes or longer training sessions.

## Considerations for future work

Although we did not find evidence that graphomotor training contributed to improvement in visual discrimination of object-like shapes beyond visual training alone, future studies could extend our experimental approach to studying if graphomotor training contributes to other aspects of learning shapes such as their matching (Ben-Ami et al., 2024) or rotation-invariant visual recognition (Li & James, 2016; Longcamp et al., 2006, 2008). Additionally, combinations with other sensory modalities during training can be further explored (e.g., haptic and auditory; Bara & Gentaz, 2011; Heller & Gentaz, 2013; Hennion et al., 2005; Martolini et al., 2020). An especially important validation is testing these effects with young children, which may differ from results with adults, given the different salience of motor versus perceptual input early in life, and the dependence of early stages of learning on real-world interactions of body and environment (Ghisio et al., 2017; James & Swain, 2011; Volpe & Gori, 2019).

A compelling avenue for future study involves extending this investigation to the *copying* of templates, and examining if this may differentially affect visual learning outcomes in comparison to the *tracing* method employed in the current study. This proposition is rooted in the insights provided by previous studies (James & Engelhardt, 2012; Li & James, 2016), which argue that the visual variability inherent in free-form handwriting could be a pivotal factor contributing to the beneficial outcomes of visual learning through graphomotor activity. To rigorously explore the comparison between copying and tracing for learning, it is essential to disentangle the specific effects of variability from other factors that distinguish these processes, such as the higher demands on memory and recall imposed by copying templates, versus the more immediate and accuracy-focused performance feedback offered by tracing them (Gonzalez et al., 2011). The research paradigm outlined in this paper lays a foundation for exploring the interplay between visual variability and cognitive processes involved in these distinct motor activities, as it allows to contrast the effect of graphomotor training (e.g., through copying) with the effect of visual training derived from mere observation of resulting products.

Another intriguing avenue is the effect of the type of learned visual stimuli on visual learning from graphomotor engagement. Since we found no advantage of tracing for visual learning with curved, closed shapes, this could be used as a baseline to test if the advantage of graphomotor engagement over visual training becomes evident when stimuli incorporate edges and/or open contours, or exhibit other grapheme-like characteristics. 

While existing research centers around the impact of *handwriting* for learning *graphemes*, the effects of *drawing* for helping students learn *shapes* has gained less attention. In educational settings, techniques involving manual reproduction of geometric shapes are commonly employed when teaching young students, but the effectiveness of this approach is not sufficiently empirically based (Gecu-Parmaksiz & Delialioglu, 2019; Hu et al., 2014; Larkin, 2016). The current empirical assessment of the relative contribution of motor and visual factors to visual learning of object-like shapes suggests that educational techniques should include practice that encourages detailed shape analysis and emphasizes the most relevant diagnostic features, either with or without manual reproduction. Future research should explore the comparative benefits of incorporating techniques for encouraging detailed shape analysis into various learning environments, to determine their impact on shape learning and retention.

## Supplementary information

Below is the link to the electronic supplementary material.

Supplementary file1 (DOCX 872 KB)

## Data Availability

The code used for running the training paradigm and for running the visual assessment are available through GitHub, respectively, at the following links: https://doi.org/10.5281/zenodo.10438; https://github.com/mit-quest/prakAI-pebbles.
