# Supplementary material for: What the visual system can learn from the non-dominant hand: The effect of graphomotor engagement on visual discrimination
Source: Mem Cognit. 2024 Nov 5;53(1):325–40. doi: 10.3758/s13421-024-01628-2 (PMC11779777; doi:10.3758/s13421-024-01628-2)
Supplement: Supplementary file 1 — Supplementary file1 (DOCX 872 KB) [file 13421_2024_1628_MOESM1_ESM.docx]

**Supplementary materials –**

**What the visual system can learn from the non-dominant hand: The effect of graphomotor engagement on visual discrimination.**

**Supplementary Methods**

**Stimuli synthesis.** To synthesize similar, yet distinct shapes, we devised a novel stepwise stimulus synthesis technique to construct parametrically adjusted stimuli, so that the similarity between them is mathematically controlled and characterized. This enabled us to determine and quantify the degree of similarity between shapes.

Stimuli were composed from combinations of basic radial frequency patterns (Wilkinson et al., 1998), which are created by modulating the radius of a circle by a sinusoidal function of the polar angle. Each stimulus was derived from a combination of five specific basic radial frequency patterns, in a manner akin to Fourier synthesis (Schmidtmann et al., 2016). The only difference between different stimuli is that one of the composing basic radial frequency patterns is oriented differently for each stimulus (Fig. S1A), with equal changes to its orientation between consecutive stimuli (e.g., equal rotation steps between each pair of consecutive stimuli 1 to 8 in Fig. 1A). This procedure resulted in a mathematically defined distance between each given pair of stimuli, which can be determined by the number of rotation steps (detailed in the main methods section). Finally, we adjusted the area of each shape to equate the perimeters of all stimuli, so that all tracing paths are rendered equal in length.

**Stimulus validation methodology.** To validate our mathematically constructed stimulus-set in terms of perceptual distance between stimuli (e.g. see Fig. 1A in the main methods section, where stimuli 1 and 2 are more confusable than stimuli 1 and 4), we tested human visual discrimination between our generated stimuli. To this end, we tested a group of 25 participants who did not participate in the main studies and underwent a delayed match-to-sample procedure identical to the one used in the main study (see the procedure of the visual assessment task in the methods section, Fig. 1B).

**Confusion matrix** (Fig. S1B). Since we had 8 stimuli, the visual assessment task yielded an 8x8 confusion matrix in which each row (i) represents a target stimulus, and each column (j) represents one of the sample shapes. The value in each cell (i,j) is the proportion of trials in which the sample stimulus j was chosen as the match for the corresponding target stimulus i, out of all trials in which it served as the target (8 per target in each run).

**Validation results**. The overall accuracy (hit rate, calculated as average of the diagonal of the confusion matrix in Fig. S1B) was near 50%, leaving enough room for improvement following training. Participants’ matches between target and sample demonstrated stepwise confusions - each target shape was confused mostly with shapes within one step of parameterization, while confusions gradually decreased with increasing parameterization steps (as illustrated by the confusion matrix in Fig. S1B and quantified per parameterization step in Fig. S1C).

**Visual assessment outcome measures calculation.** After validating that our stimuli indeed create the perceptual effect we expected, we were able to use their mathematical properties to create two visual discrimination measures for our main studies. To this end, we used the confusion matrix data to calculate:

(1) **Accuracy**. The proportion of correct matches (hits) is the average of the diagonal of the confusion matrix described above.

(2) **Relative** **distance error** quantification of the misses. To calculate this measure, the values in each cell (i,j) of the original confusion matrix were multiplied by the distance of the target stimulus (i) from the match stimulus (j), creating a distance error matrix. The distance of a target stimulus from itself (hit trials) is 0. The average of all cells in this distance error matrix is taken as the *relative distance error* of a given participant.

*
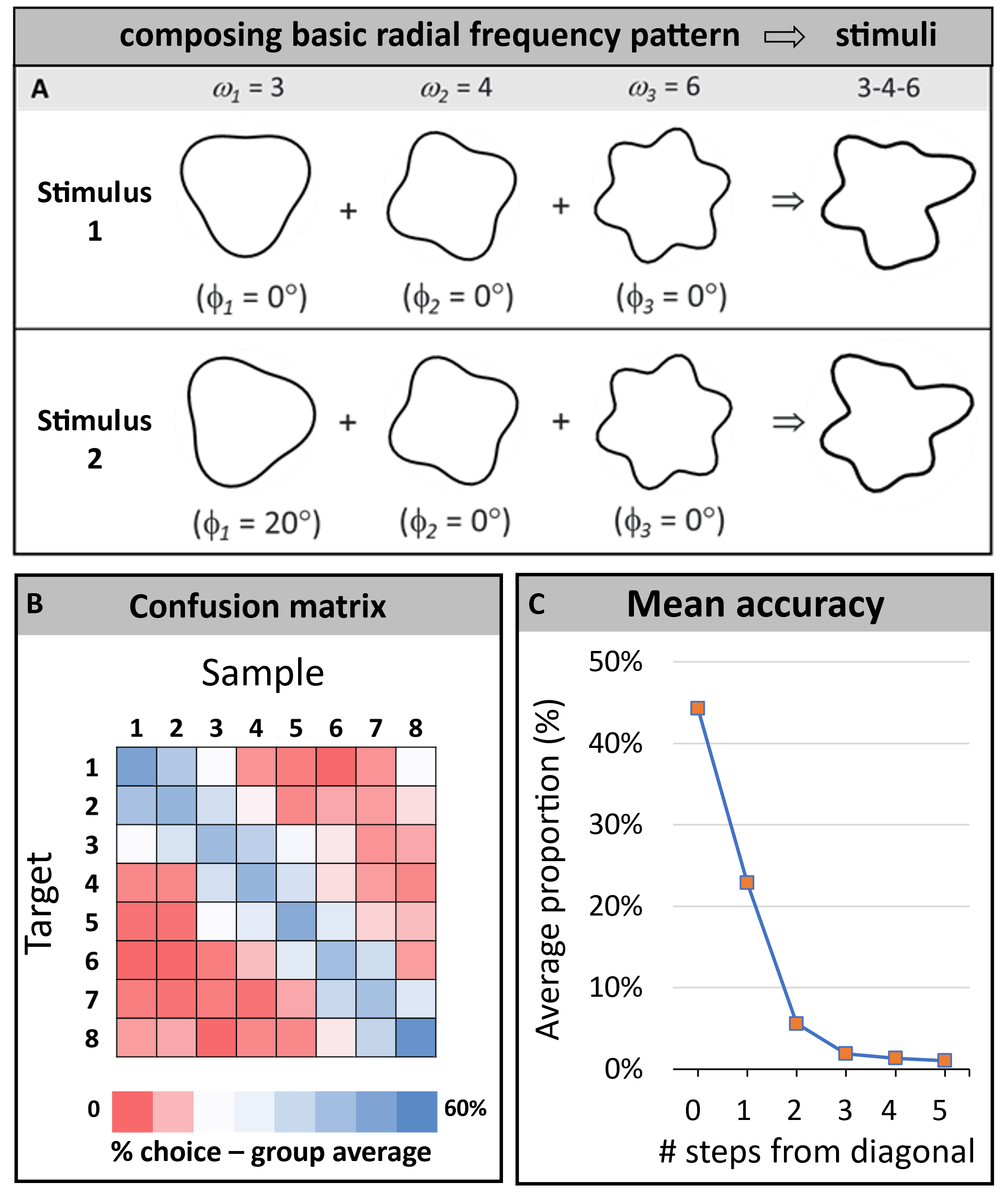
Fig. S1.* ***A. Stimuli construction methodology.*** *Illustration of the construction of two example stimuli (right) from three basic radial frequency patterns (left). Note that we used five radial patterns for construction of our final stimuli. Both example stimuli are constructed from the same basic patterns - the frequency of each basic pattern (ω, corresponding with the number of bulges) is denoted on the top, and its phase angle (ɸ, corresponding with the pattern’s rotation) is denoted below it. The* ***only difference*** *between the two stimuli is the rotation of the first composing pattern (note that ɸ_1_ of Stimulus 1 is 0° and of Stimulus 2 is 20°).*

***B. Mean confusion matrix*** *(n = 25) obtained from the visual assessment delayed match-to-sample task (see methods section and Fig. 1B). Each cell in the matrix represents the proportion of trials that a target stimulus (row number) was matched to a specific sample stimulus (column). The proportion in each cell is calculated out of 8 trials in which a specific stimulus served as target. The diagonal represents hit trials and off-diagonal miss trials. Note the higher proportion of hit trials (diagonal) and that most confusions are near the diagonal (i.e., parametrical neighbors). Also note that participants are not at ceiling performance – leaving room for training-induced improvement.*

***C. Mean confusion as a function of distance from target stimulus.*** *In the highest proportion of trials, the target was matched with itself (hit; # of steps = 0). The proportion of trials confused with the target monotonously decreases as the parametric distance between the target and the sample increases.*

***Supplementary Results***

**Experiment 2 - Visual Discrimination Performance Analysis.**

In experiment 2, we found differences in visual discrimination baseline performance between the two groups. Thus, the most informative analyses of group effects are baseline-adjusted measures of individual subjects, which are not affected by these pre-training group differences.

Below we provide the (less informative) statistical analysis of non-adjusted measures (see Figs. 4A and 4D). Time effects were examined using a mixed design ANOVA on the original measures, with the three testing points as a within-participant factor (Time), and the training groups (Graphomotor non-dominant, Graphomotor dominant) as a between-participant factor (Group). We conducted this analysis separately for the accuracy (Fig. 4A) and distance error measures (Fig. 4D).

***Accuracy (Fig. 4A).***

**Improvement across time.** The analysis of visual discrimination accuracy revealed a significant increase in visual discrimination accuracy after training, and a persistence of this gain in performance one week later. This was indicated by the significant effects of Time on performance (F(2,36)=51.05, p<.001, *η^2^_p_*= 0.74; Mean ± SD of accuracy: pre-training 45.70% ± 8.68%, post-training 66.38% ± 12.14%, retention 66.93% ± 13.96%). Post hoc testing using the Bonferroni correction indicated a significant increase in accuracy between pre-training and post-training sessions (Mean improvement of 21.5%, SD = 12.75%, p<.001, d = -1.92) with this improvement maintained at the retention phase (Mean improvement between pre-training and retention of 21.9%, SD = 11.11%, p<.001, d = -1.95). We did not detect a significant difference between post-training and retention accuracy (Mean difference 0.4%, SD = 8.18%, p=1, d = -0.04).

**Difference between groups.** Although the t-test analysis examining pre-training group differences indicated significant differences between visual discrimination prior to training, the main effect of Group in the ANOVA failed to reach significance. No significant differences were found between the groups who trained with different hands in terms of accuracy, as reflected by the insignificant effect of Group (F(1,18)=3.73, p=0.07, *η^2^_p_* = 0.17; Mean ± SD of accuracy: Graphomotor non-dominant 62.2% ± 12%, Graphomotor dominant 57.23% ± 13.12%), and insignificant interaction between Time and Group (F(2,36)=0.32, p=0.73, *η^2^_p_* = 0.02).

**Bayesian analysis.** To further quantify the findings from null hypothesis significance testing, we conducted a Bayesian mixed design ANOVA, which determined that the data were best represented by a model that includes only the Time factor. The Bayes factor BF_01_ for the Time model was 4.13*10^-10^, indicating decisive evidence in favor of this model when compared to the null model, and confirming the significant effect of Time on accuracy improvement previously found. When comparing the effect of Group to the null model, the Bayes factor indicated anecdotal evidence in favor of the null model over a model with an effect of Group (BF_01_ = 1.12). The Bayes factor BF_01_ for the Time X Group interaction model was 4.06, indicating moderate evidence in favor of the null model.

***Distance Error (Fig. 4D).***

**Improvement across time.** The analysis of distance error revealed a significant decrease in error after training, and a persistence of this error reduction one week later. This was indicated by the significant effect of Time on performance (F(1.34,25.12)=93.06, p<.001, *η^2^_p_*= 0.84; Greenhouse-Geisser corrected due to Mauchly’s test of sphericity showing a significant difference in variation between the group differences (W(2) = 0.5, p= 0.003; Mean ± SD of distance error: pre-training 0.74 ± 0.26, post-training 0.30 ± 0.17, retention 0.35 ± 0.15). Post hoc testing using the Bonferroni correction determined that distance error was reduced between pre- and post-training (Mean reduction of 0.38, SD = 0.18, p<.001, d = 2.15) and this was maintained at retention testing a week after training completion (Mean reduction between pre-training and retention of 0.4, SD = 0.16, p<.001, d = 2.25). No significant difference was observed between post-training and retention performance (Mean difference -0.02, SD = 0.08, p=1, d = 0.1).

**Difference between groups.** No significant differences in terms of distance error were found between the groups who trained with different hands, as reflected by the insignificant effect of Group (F(1,18)=3.84, p=0.06, *η^2^_p_* = 0.18; Mean ± SD of distance error: Graphomotor non-dominant 0.42 ± 0.14, Graphomotor dominant 0.55 ± 0.19) and the insignificant interaction between Time and Group (F(2,36)=1.07, p=0.33, *η^2^_p_* = 0.06).

**Bayesian analysis.** To further quantify the findings from null hypothesis significance testing, we conducted a Bayesian mixed design ANOVA, which determined that the data were best represented by a model that includes both the Time factor and the Group factor. The Bayes factor BF_01_ for the Time model was 1.64*10^-13^, indicating decisive evidence in favor of this model when compared to the null model, and confirming the significant effect of Time on accuracy improvement previously found. When comparing the effect of Group to the null model, the Bayes factor indicates anecdotal evidence in favor of the null model over a model with an effect of Group (BF_01_ = 1.16). The Bayes factor BF_01_ for the Time X Group interaction model was 2.4, indicating anecdotal evidence in favor of the null model.
